# Role of miRNA-1 and miRNA-21 in Acute Myocardial Ischemia-Reperfusion Injury and Their Potential as Therapeutic Strategy

**DOI:** 10.3390/ijms23031512

**Published:** 2022-01-28

**Authors:** Eranthi Jayawardena, Lejla Medzikovic, Gregoire Ruffenach, Mansoureh Eghbali

**Affiliations:** Division of Molecular Medicine, Department of Anesthesiology, David Geffen School of Medicine at UCLA, BH-550 CHS, Los Angeles, CA 90095, USA; ejayaw001@g.ucla.edu (E.J.); lmedzikovic@g.ucla.edu (L.M.); GRuffenach@mednet.ucla.edu (G.R.)

**Keywords:** miRNA-1 (miR-1), miRNA-21 (miR-21), cardiovascular disease, myocardial ischemia-reperfusion injury, myocardial infarction, coronary artery disease, cardioprotection, cardiomyocytes, fibroblasts, immune cells

## Abstract

Coronary artery disease remains the leading cause of death. Acute myocardial infarction (MI) is characterized by decreased blood flow to the coronary arteries, resulting in cardiomyocytes death. The most effective strategy for treating an MI is early and rapid myocardial reperfusion, but restoring blood flow to the ischemic myocardium can induce further damage, known as ischemia-reperfusion (IR) injury. Novel therapeutic strategies are critical to limit myocardial IR injury and improve patient outcomes following reperfusion intervention. miRNAs are small non-coding RNA molecules that have been implicated in attenuating IR injury pathology in pre-clinical rodent models. In this review, we discuss the role of miR-1 and miR-21 in regulating myocardial apoptosis in ischemia-reperfusion injury in the whole heart as well as in different cardiac cell types with special emphasis on cardiomyocytes, fibroblasts, and immune cells. We also examine therapeutic potential of miR-1 and miR-21 in preclinical studies. More research is necessary to understand the cell-specific molecular principles of miRNAs in cardioprotection and application to acute myocardial IR injury.

## 1. Introduction

Coronary artery disease remains the leading cause of death for both men and women of most ethnicities in the United States [1,2]. Roughly 659,000 Americans die from heart disease annually: accounting for one in every four deaths [3]. Acute myocardial infarction (MI) is characterized by decreased blood flow to the coronary arteries, resulting in pathological states in the myocardium, including mitochondrial dysfunction and, if continued, cardiomyocytes death [4]. The most effective strategy for treating an MI is early and rapid myocardial reperfusion via thrombolytic agents or percutaneous coronary intervention (PCI) [5]. However, restoring blood flow to the ischemic myocardium can induce further damage, known as ischemia-reperfusion (IR) injury. Mitochondria, the main energy reservoirs of cells, play a critical role in cellular function and vitality in the heart. Cells of ischemic hearts switch from aerobic respiration to anaerobic glycolysis for ATP production. As a result, intracellular levels of calcium and sodium rise and, if continued, leads to prolonged ischemia [4]. When reperfusion occurs in an ischemic heart, these ischemic conditions worsen to drive mitochondrial damage, ensuing cell death [4]. As a result, dead tissue accumulates in the myocardium and contributes to the increase in the infarct size over time [4]. Thus, myocardial reperfusion is a “double edge sword” due to its paradoxical nature to protect as well as damage the heart [6].

Novel therapeutic strategies are critical to limit myocardial IR injury and improve patient outcomes following reperfusion intervention. The ischemic mediators of myocardial reperfusion injury operate within the first few minutes of myocardial reperfusion, offering a tight therapeutic window for reducing MI size in patients undergoing PCI. A clear understanding of IR injury mechanisms is needed to overcome barriers to developing a safe and effective therapeutic agent. microRNAs (miRNAs) are promising candidates to modulate the molecular and cellular processes involved in IR injury. miRNAs are small non-coding RNA molecules (~22 nucleotides in size) that have been implicated in attenuating IR injury pathology in pre-clinical rodent models [4]. The main function of miRNAs is to promote degradation and inhibit the translation of protein-coding genes by annealing to target mRNAs [7,8]. Each miRNA may modulate 10–100 s of mRNA genes and thus can control multiple cellular pathways at once. Additionally, miRNAs can exist as clusters in the mammalian genome and be transcribed as polycistronic primary transcripts. The purpose of the miRNA clusters is to potentially regulate every aspect of cellular function, including growth, development, cell death, among others [9]. Overall, these miRNAs have a profound impact on cardiac pathology and, when dysregulated, contribute to the disease, as is the case in acute myocardial IR injury [10,11,12]. miRNAs are favorable therapeutic strategies as they can be targeted with high affinity and specificity. In addition to emerging as potential therapeutic regulators of IR injury, miRNAs can also be excreted from cells into the circulation and act as versatile endogenous signals [13,14]. Thus, miRNAs could serve as biomarkers to detect and determine risk profiles for IR injury in vulnerable MI patients [15,16,17]. 

miRNAs are extensively involved in molecular pathways of cardiac diseases related to IR injury, including arrhythmia triggered after an MI [18] as well as atherosclerosis or obesity, which predisposes patients to MI [13]. Thus, miRNAs dysregulated in the whole disease process (atherosclerosis, MI and IR injury) would make more attractive therapeutic targets than miRNAs only dysregulated in one aspect of the disease. By this rationale, for this review, an independent analysis using HMDD (the Human microRNA Disease Database, V3.2) [19,20] identified miRNA-1 (miR-1) and miRNA-21 (miR-21) as top hits of multiple cardiovascular disease states in the context of IR injury. 

Cardiac cell types can differentially express miRNAs during disease onset and progression. Several databases, including the previously mentioned HMDD, have successfully mapped miRNA expression profiles to specific cell types and identified their downstream targets. The purpose of mining these big data tools is to identify miRNAs that are differentially expressed in human cardiovascular disease (CVD) and also discover any shared signatures among related diseases. Despite many research efforts thus far, it remains unclear whether any miRNAs protect the heart from IR injury in humans. Here, we will highlight the most recent molecular perspectives of the mechanistic and therapeutic roles miR-1 and miR-21 play in IR injury and related cardiac diseases. 

## 2. miRNAs: A Link between IR Injury and Other Cardiac Diseases in Humans

Coronary artery disease is the ultimate culprit of MI and subsequent IR Injury. Injury resulting from reperfusion accounts for half of the final MI damage in acute MI [21] and contributes to many clinical complications, including arrhythmias and myocyte death, a hallmark of IR injury [4,22]. These cardiac-related complications in patients with severe IR injury are becoming increasingly common. Additionally, the adverse effects of IR injury-related complications on the heart can be irreversible and life-threatening [22], resulting in heart failure and patient’s death later in life [23,24]. The exact pathophysiological mechanisms of IR injury are not fully known [25,26,27], and this continues to be a foreseeable challenge for clinicians [28,29]. 

miRNAs have emerged as powerful regulators of gene expression and are involved in many cardiovascular diseases. In IR injured hearts, several miRNAs are aberrantly expressed [1] and regulate the disease progression. While several dysregulated miRNAs are unique to IR injury, a few of those overlap with other cardiac diseases in humans and murine models [13]. The miRNAs were independently expressed in each disease state. With more research, we can discern whether the overlap in miRNAs may indicate that patients with coronary artery disease (CAD) may develop IR injury. Most likely, miRNAs dysregulated in the entire disease process, leading to IR injury (CAD, MI, and IR injury), make powerful therapeutic targets than miRNAs only dysregulated in one aspect of disease (IR injury only). Thus, miRNAs common between IR injury and early stages of coronary artery disease, and even MI and other cardiac diseases, could potentially serve as powerful therapeutic targets of protection against myocardial reperfusion injury. 

**Figure 1:**
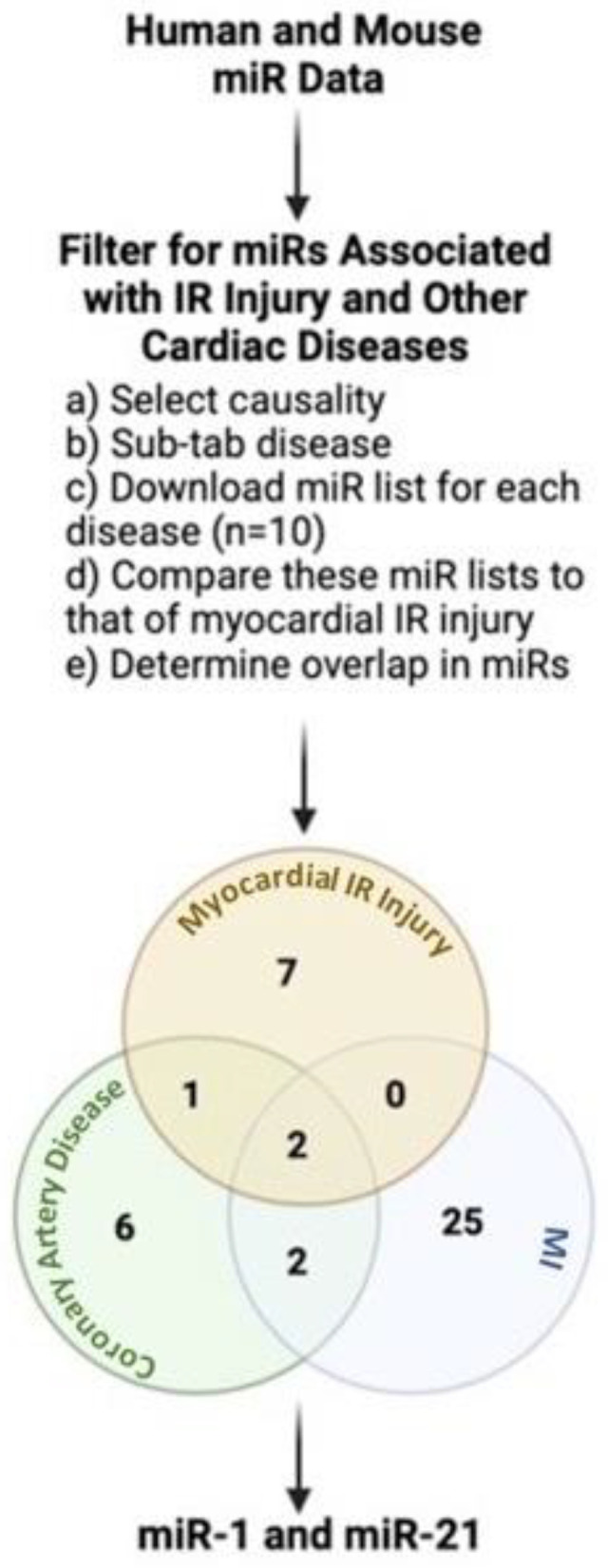
HMDD pipeline to filter for miRs dysregulated in myocardial IR injury and other cardiac diseases. miR-1 and miR-21 are dysregulated in myocardial IR injury, CAD, and MI. The findings from the HMDD analysis for miR-1 have been modified with a literature search to include CAD and myocardial IR injury. IR: ischemia-reperfusion injury; MI: myocardial infarct. Created with BioRender.com.

For this review, a publicly available miRNA evidence-supported database, HMDD (the Human microRNA Disease Database, V3.2) [19,20], was used to identify miRNAs associated with IR injury and other cardiac diseases in humans and animal models. After selecting the causality tab, the disease sub-tab in HMDD guided with narrowing the search to cardiac diseases, which shared dysregulated miRNAs common with IR injury. The search terms were coronary artery disease, coronary atherosclerosis, atherosclerosis, acute myocardial infarction, myocardial infarction, cardiac fibrosis, arrhythmia, cardiac myocyte injury, ischemia-reperfusion injury, and myocardial ischemic-reperfusion injury. The list of miRs for each disease state was downloaded and compared to that of myocardial IR injury to determine any overlap in miRs. CAD and MI were the only two disease states with miRs common with myocardial IR injury. When these disease states were compared together, miR-21 was the only miR common between all three disease states (Figure 1). Furthermore, the HMDD analysis revealed that miR-1 is a mediator of similar disease states as miR-21 (MI and myocardial IR injury). While the HMDD analysis did not find miR-1 to be dysregulated between CAD and myocardial IR injury, the literature search highly supports that miR-1 is dysregulated in these two disease states [30,31,32,33,34]. 

### 2.1. Role of Cardiac miR-1 in IR Injury

miR-1 is a cardiac abundant miR [35] tightly associated with IR injury [30]. The dysregulation of cardiac abundant miR-1 has been repeatedly shown to be injurious to cardiomyocytes [36,37,38] and the whole heart [31,39,40]. miR-1 is downregulated in response to myocardial IR injury in the heart tissues of rodents [31,32,33] as well in cardiomyocytes that underwent hypoxia-reoxygenation [31,34]. The miR-1 levels decrease significantly with prolonged reperfusion time in MI rats or reoxygenation time in H9C2 cells [31]. Additionally, miR-1 is downregulated in heart tissues of infarcted human hearts collected by post-mortem autopsies [41,42]. After the infarcted hearts were separated into groups based on MI duration, miR-1 is downregulated only in patients with greater than one or less than seven days after MI [41]. Thus, the results are indicative of temporal changes in miR-1 expression during IR injury or wound healing that follows MI. Yet, miR-1 is upregulated in the remote myocardium when compared to healthy or infarcted adult hearts [43]. However, in another study, Pan et al. showed miR-1 transgenic mice subjected to IR injury had worse cardiac injury compared to wild-type mice and treatment with LNA-antimiR-1 attenuated the IR injury [30]. Overall, the study by Pan et al. established that miR-1 plays an important role in cardiac injury, and reducing miR-1expression effectively mitigates the myocardial injury. These studies demonstrate mixed conclusions about the effects of miR-1 on IR injury (Table 1), most likely explained by differences due to reperfusion times or disease models. 

miR-1 in circulation shows optimal stability and protection against degradation by RNAse activity in the body [17]. The stability of miR-1 has stirred interest in its use as a potential biomarker for the diagnosis and prognosis of CVD, including myocardial IR injury [17]. While reduced levels of miR-1 exist in heart tissue during IR injury, levels of circulating miR-1 were significantly increased in acute MI patients receiving PCI intervention [44]. In a comparative study, aged mice exposed to MI had higher circulating miR-1 than young mice, and this was linked to severe cardiac remodeling [45]. Kelm et al. concluded that the significantly elevated miR-1 could be a predictive biomarker of myocardial injury in high-risk older human subjects [45] Overall, miR-1 was released from heart to blood stream in ischemia or post-MI and this may indicate an adaptive mechanism of heart to ischemia considering elevated circulating miR-1 worsens IR injury. Yet, miR-1 is not used in therapies or as a biomarker of cardiac injury in clinical trials. This could be due to the fact that the role of miR-1 as a biomarker is still highly debated despite reports that support the associations between increased levels of miR-1 and IR injury [45]. The difficulty to translate research findings to clinical use could be due to lack of properly-sized sample size, inconsistent experimental protocols and also, age and apparent sex-specific differences. All of these limitations need to be addressed first prior to using miR-1 or any miRNA in clinical practice to treat IR injury and its clinical complications. 

IR injury may induce arrhythmia, which is common in patients with post-MI treatment such as thrombolytic therapy, PPCI, and cardiac surgery [46]. IR-induced arrhythmia typically occurs within the first 20 min of reperfusion [46]. The same pathophysiology that exists in IR injury, e.g., apoptosis and mitochondrial dysfunction, drives many forms of arrhythmias, including the main type, accelerated idioventricular rhythms [47]. Thus, the two conditions complement each other and, together, contribute to larger infarct size, meaning more myocyte death and decreased ventricular functioning [47,48,49,50,51,52,53]. Notably, studies support that reperfusion without arrhythmia has a smaller infarct size regardless of the same initial areas at risk [47,48,49,50,51,52]. To date, the pathophysiology that explains IR-induced arrhythmias remains to be somewhat unclear.

Several studies have implicated that miRNA-1 drives arrhythmogenesis by altering ion channels and proteins associated with the heart’s electrical activity in cardiac diseases [54,55,56], including ischemia-reperfusion injury [18]. Connexin 43 (Cx43), a major gap junction channel in the heart, is involved in regulating cardiac conduction and, when dysregulated, results in ventricular arrhythmias [57]. Studies have indicated that both IR injury and IR-induced arrhythmia are followed by changes in Cx43 expression [58]. Specifically, Bian et al. reported that Cx43 was down-regulated in response to IR injury [59], and down-regulation of miRNA-1 can prevent the decrease of Cx43, ultimately protecting the heart from IR injury [18]. Iroquois homeobox domain 5 (Irx5), another target of miRNA-1, creates the cardiac repolarization gradient by blocking potassium voltage-gated channel subfamily D member 2 (KCND2) [57]. KCND2 encodes the subunit contributing to pore for Ito (transient outward potassium current), which repolarizes action potential phase I [57]. IR model of MI has a reduced Ito current and a persistent action potential duration. Myers et al. found that introducing miRNA-1 to animals stopped the increase in Irx5 expression and decreased in KCND2 levels, normalizing Ito and action potential duration [60]. Taken together, the mechanistic link between miRNA-1 with IR-induced arrhythmias supports the therapeutic potential of miRNA delivery post-MI in IR-induced MI animal models. 

### 2.2. Role of Cardiac miR-21 in IR Injury

Major et al. delved into sourcing HMDD for any miRNAs that were associated with CVD by relying on IR injury and its related diseases as a model [13]. Key findings from this study revealed that a single miRNA, hsa-miR-21, causally links ischemia reperfusion injury in the heart to coronary artery disease, stroke and obesity [13]. Moreover, hsa-miR-21 was the only miRNA consistently detected in circulation for all four disease states, and target analysis indicated that this miRNA can modulate several inflammatory and apoptotic genes (evident in pathways of IR injury and related cardiac diseases) [13]. The overall study proposes that hsa-miR-21 is a robust biological molecule for selective cardioprotective agents and biomarker discovery.

miR-21 is expressed at high levels in CVD and its major risk factor, obesity. However, the protective effects of miR-21 in the heart are debated due to varying cardiac disease models and tissue types. While upregulated miR-21 protects hearts from ischemic injury in mice [61,62], (Table 1) the same miRNA is detrimental during vascular injury induced in male rats using a carotid artery balloon injury model [63] and leads to cardiac hypertrophy [64,65]. One possibility is that acute upregulation of miR-21 is cardioprotective against ischemia injury, whereas continuous expression of this miRNA may be related to cardiac hypertrophy and obesity (risk factors for MI or stroke). 

Several studies have explored molecular mechanisms of miR-21 modulation in CVD pathology by finding accepted mRNA targets. Endothelial progenitor cells (EPCs) are crucial to maintaining vascular health, and injury to this cell type is strongly linked to CVDs, specifically atherosclerosis [66]. miR-21 prevents EPC proliferation by activating TGF β signaling pathway through downregulation of WWP1 [67]. These findings may help design molecular approaches to improve the vitality of EPCs for future therapeutic applications. Ample evidence supports the idea that miR-21 is dysregulated even prior to IR injury, when atherosclerotic plaque first develops in the arteries. Major et al. identifies pro-inflammatory IL-12A as a core target of hsa-miR-21-5p involved in arterial stiffness that contributes to early atherosclerosis in humans [13,68]. Cao et al. report miR-21 to be significantly upregulated in atherosclerotic plaque compared to controls, whereas Jag1, a proven target of miR-21, is significantly downregulated [69]. The authors claim that selective inhibition of this miRNA may provide insight to prevent atherosclerotic plaque from progressing. miR-21 is an excellent target to prevent not only IR pathology, but also the root of this disease—atherosclerotic plaque in arteries.

If left untreated or undetected, atherosclerotic plaques in the coronary arteries are prone to rupture and result in acute MI. Novel repair mechanisms involving extracellular vesicles are being recognized to protect against MI. Inhibition of miR-21 reduced the cardioprotective effects of extracellular vesicles in attenuating cardiomyocyte death [70]. miR-21 protects cardiomyocytes against MI and reactive oxygen species (ROS)-induced injury by targeting programmed cell death 4 (PDCD4) gene [70,71]. A repair strategy targeting extracellular vesicles can serve as a potential therapy for acute MI and ischemia-reperfusion injury. 

Another study reports rno-miR-21-5p as a potential biomarker of inflammation in the heart after acute drug-induced cardiac injury in rats [72]. When looking at the top target pathways of 3′ mature miR-21 strand [13], the target analysis suggests MAP2K4 and MAP3K1 as key drivers of cardiac hypertrophy [73,74]. Per these studies, miR-21 associations with IR injury and related cardiac diseases are well-supported by direct experimental evidence. Thus, precise regulation of miR-21 function and their subsequent target genes could unravel the mechanism of IR pathology and offer effective therapies for multiple complex.

**Table 1:** Summary of most relevant cardiac injury studies related to miR-1 and miR-21 cardiac diseases.

miR	Findings	Function	Therapeutic Potential	Reference
miR-1	↓ miR-1 in rodent hearts in response to IR injury.	↑ Bcl-2 after IR (HR) injury	miR-1 inhibition ↑ Bcl-2 and ↓ IA/AAR and cell apoptosis after IR(HR) injury	[31]
	↑ miR-1 in rodent hearts in response to IR injury	↓ Bcl-2 and Cnx43 after IR (HR) injury	miR-1 mimic ↓ Bcl-2 and Cnx43 in H9C2 cells subjected to HR injury; Telmisartan ↑ Bcl-2 and Cnx43 and ↓ miR-1 after IR (HR) injury	[32]
	↑ miR-1 in rodent hearts in response to IR injury or MI	↓ KCNJ2/Kir2.1 and GJA1/Cx43 in ischemic myocardium	miR-1 overexpression ↓ KCNJ2/Kir2.1 and GJA1/Cx43 after MI; sEHIs reversed the effects	[33]
	↓ miR-1 in infarcted human hearts in response to MI	N/A	N/A	[41,42]
	↓ miR-1 in H9c2 cells in response to HR	↑ Bcl-2 after IR (HR) injury	miR-1 inhibition ↑ Bcl-2 and ↓ IA/AAR and cell apoptosis after IR(HR) injury	[31]
	↑ miR-1 in neonatal cardiac myocytes in response to HR	↑ apoptosis and ↓ Bcl-2 after HR injury	miR-1 mimic ↑ apoptosis and ↓ Bcl-2 in neonatal rat cardiomyocytes subjected to HR injury; H2S reverses the effects	[34]
	↑ miR-1 in remote myocardium compared to infarcted zone or healthy hearts in infarcted human hearts	N/A	N/A	[43]
	↑levels of serum miR-1 after acute MI in pigs and humans	N/A	N/A	[44]
	miR-1 overexpression worsened cardiac I/R injury in transgenic mice	↑ LDH, CK levels, caspase-3 expression, apoptosis and cardiac infarct area after IR (HR) injury	miR-1 overexpression exacerbate IR (HR) injury by ↑ LDH, CK levels, caspase-3 expression, apoptosis and cardiac infarct area	[35]
	miR-1 inhibition protects against IR (HR) injury in rodents and cardiomyocytes	↑ LDH, CK levels, caspase-3 expression, apoptosis and cardiac infarct area after IR (HR) injury	LNA-antimiR-1 attenuated IR (HR) by ↑ PKCε and HSP60	[35]
	miR-1 inhibition protects against IR (HR) injury in rodents and H9c2 cells	↑ Bcl-2 after IR (HR) injury	miR-1 inhibition ↑ Bcl-2 and ↓ IA/AAR and cell apoptosis after IR(HR) injury	[31]
miR-21	↓ miR-21 in infarct areas of mouse IR model	↑ apoptosis and PDCD4, Bax/Bcl-2 and cleaved caspase-3/caspase-3 ratio after IR injury	miR-21 mimics ↓ apoptosis by inhibiting PDCD4 in cardiomyocytes subjected to OGD/R	[70]
	diverse time-dependent changes in circulating miR-21 in post-MI patients	N/A	N/A	[13]
	miR-21 protected cultured cardiac myocytes against HR-induced apoptosis via its target PDCD4	↑ apoptosis and PDCD4, Bax/Bcl-2 and cleaved caspase-3/caspase-3 ratio after IR injury	miR-21 mimics ↓ apoptosis by inhibiting PDCD4 in cardiomyocytes subjected to OGD/R	[70]
	miR-21 protected cultured cardiac myocytes against HR-induced apoptosis via its target PDCD4	↑ apoptosis in cardiomyocytes treated with H2O2	pre-miR-21 ↓ H2O2-induced apoptosis of cardiomyocytes; overexpression of PDCD4 inhibited pre-miR-21 mediated protective effect	[71]

Abbreviations: IR: ischemia-reperfusion; HR: hypoxia-reoxygenation; MI: myocardial infarction; PDCD4: programmed cell death protein 4; Bcl-2: B-cell lymphoma 2; IA/AAR: infarct area/area at risk; Cnx43: connexin43; KCNJ2/Kir2: potassium voltage-gated channel subfamily J member 2 (encoding potassium channel subunit Kir2.1); GJA/Cn43: gap junction protein alpha 1 (encoding connexin43); sEHIs: soluble epoxide hydrolase inhibitors; H2S: hydrogen sulfide; LNA-antimiR-1: locked nucleic acid modified oligonucleotide against miR-1; PKCε: protein kinase C epsilon; HSP60: heat shock protein 60; OGD/R: oxygen-glucose deprivation and reperfusion.

## 3. Role of miR-1 and miR-21 in Different Cell Types of IR Injured Hearts 

### 3.1. Single-Cell Sequencing Data on IR Injury

Existing therapies to salvage the myocardium following an MI mainly focus on revascularizing the blocked artery. However, the adult human heart cannot fully heal or regenerate after cardiac injury, which contributes to the irreparable loss of cardiomyocytes [75]. With fewer myocytes, the injured heart remodels aberrantly and fails to contract efficiently [76,77]. Many cells, including fibroblasts and endothelial cells, change phenotype, whereas neutrophils, macrophages, lymphocytes are recruited to sites of myocardial injury to jumpstart the healing process [78,79,80,81]. Two physiological functions dictate a healthy heart: the ability for cells to communicate and coordinate with each other [76]. Overall, four related phases highlight cardiac remodeling post-MI and involve all these cells: inflammatory, proliferative, maturation, and remodeling phases [77]. The first phase, inflammatory, is triggered by a loss of myocytes at the site of infarct. In response, endothelial cells would enhance vascular permeability to immune cells that assist with removing dead cells in the infarcted site. In the proliferative phase, inflammatory responses fade as macrophages switch phenotype and repair mechanisms become dominant. Next, fibroblasts and endothelial cells multiply, lay down collagen, and create a microvascular bed in the dead myocardium. In the maturation phase, activated fibroblasts replace the damaged heart muscle with the scar by secreting ECM proteins. Lastly, the damaged heart undergoes pathological remodeling.

Recently, high throughput single-cell RNA sequencing (scRNAseq) reveals differentially expressed genes in cardiac cell types of IR injured mammalian hearts [82]. From such data, the ability to study the role of various types of cells during wound healing response following IR is possible. Additionally, these data can help link changes in gene expression to changes in cell function and explain cellular interactions relevant for cardiac repair. 

Molenaar et al. used a FACS-based scRNA-seq approach to outline cellular distribution, biological role and crosstalk following IR injury to the adult heart [83]. Neutrophils were present in early times of cardiac injury, while fibroblasts and macrophages were detected mid-time point and declined as injury continued long-term [83]. Furthermore, functional data using differentially expressed genes suggest a switch in fibroblasts and macrophages to anti-inflammatory and pro-repair/angiogenic types. These cellular switches were consistent with recent bulk-RNA sequencing performed on the whole heart tissues [84,85]. In both studies, [83,84] the authors found that the transcriptional profile of macrophages was a continuum—from day 0 to day 7—from a more pro-inflammatory phenotype toward a more pro-resolving phenotype. In addition, both studies highlight the need to redefine our M1/M2 macrophages phenotypic definition as they both found that the arginase—an archetypal marker of M1 phenotype—is up-regulated (900-fold) at day 1. Thus, these studies challenge our conventional view of an almost pure M1 macrophages population at early stage moving toward an exclusive M2-macrophages later on, as well as our current definition of M1/M2 macrophages phenotype. Although previously studied at single-cell scale [86,87,88,89], a detailed analysis of how exactly these cell types behave during cardiac repair will prove necessary to develop impactful treatments for IR injury. 

### 3.2. Role of miR-1 and miR-21 in Cardiac Cell Types Post-MI

#### 3.2.1. Cardiomyocytes

Cardiomyocytes, high energy-demanding cells, give the ability for the heart to contract and perform mechanical pumping. In IR injury, cardiomyocytes become vulnerable to death following a lack of blood supply [90]. A major approach is to therapeutically target miRNAs that prevent cardiomyocyte cell death after ischemic-reperfusion stress. 

**Figure 2:**
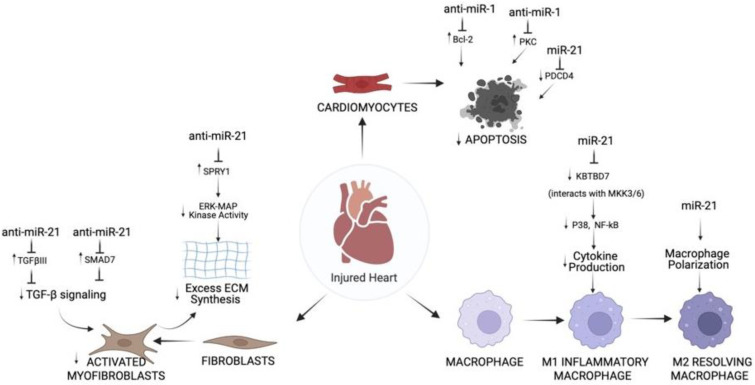
Overview of Action of miR-1 and miR-21 on Cardiomyocytes, Fibroblasts, and Immune Cells in Hearts Subjected to I/R Injury. In cardiomyocytes, inhibition of miR-1 prevents apoptosis via Bcl-2 and PKC, whereas miR-21 inhibits apoptosis via PDCD4. In cardiac fibroblasts, inhibition of miR-21 prevents TGF-β signaling and ECM synthesis via SMAD7, TGFβIII, or SPRY1. In immune cells, miR-21 regulates multiple aspects of macrophage function, including inhibition of cytokine production and activation of macrophage polarization through P38, NF-kB. Created with BioRender.com.

Liu et al. explain the effects of miRNAs on cell types and expand on molecular roles of miRNAs that control cell-specific actions [76]. miR-1 and miR-21 were identified to mediate components of apoptosis pathways in injured cardiomyocytes during MI. miR-1 directly inhibits the anti-apoptotic protein Bcl-2 in cardiomyocytes of the IR injury rat model [38]. The data reveal that miR-1 is important to regulate cardiomyocyte apoptosis, which entails post-transcriptional repression of Bcl-2 [38]. Another pathway implicated in cardiomyocyte apoptosis include anti-apoptotic protein kinase C epsilon (PKCε) [76]. In cardiomyocytes, miR-1 worsened ischemia-reperfusion injury in in vivo mouse models [30]. Pan et al. identified PKCε and HSP60 as suppressed miR-1 molecular targets in the cardiac injury pathways involving apoptosis [30]. In summary, the study showed that miR-1 is a causal miRNA for cardiac injury and systemic LNA-antimir-1 therapy prevents this condition [30]. One last notable pathway mediating apoptosis involves the molecule programmed cell death 4 (PDCD4), which increases in expression during apoptosis and functions as proapoptotic inhibitor of genes [76]. miR-21 directly inhibits PDCD4 signaling and prevents apoptosis in cardiomyocytes during MI [70,71]. Altogether, the therapeutic application of miR-1 and miR-21 in heart disease related to ROS such as MI and myocardial IR injury is to prevent cardiomyocyte cell death (Figure 2). 

#### 3.2.2. Fibroblasts

Cardiac fibroblasts become activated following MI and differentiate into myofibroblasts, which form scars that resist ventricular wall rupture. The continuous activation of cardiac fibroblasts, proliferation, and ECM deposition after MI results in pathological cardiac fibrosis, which can worsen the injury and elicit heart failure [76]. miRNAs are a therapeutic approach that can reverse the activated phenotype to dampen cardiac fibrosis.

Transforming growth factor-β (TGF-β) is a key regulator in fibroblast repair of heart after MI [91], and signaling rely on proteins such as decapentaplegic homologs (SMADs). TGF-β decreases MMPs [92] and, at the same time, increases the generation of collagen type 1 and 3, leading to ECM production [93]. TGF-β receptor III (TGFβIII) is a known negative regulator of TGF-β signaling [94]. Liang et al. explain a reciprocal loop in MI-induced cardiac fibrosis in mice by which TGF-β upregulates miR-21, which subsequently downregulates TGFβIII [95]. miR-21 inhibition of TGFβIII increases collagen secretion by high TGF-β release and phosphorylated-Smad3 [95]. The study proposes miR-21 and TGFβIII pathway as a potential target to prevent and treat myocardial remodeling after MI. TGF-β signaling is inhibited by SMAD-7; thus, anti-miRNAs against this SMAD can prevent fibrosis. In a study, miR-21 regulates myocardial fibrosis after MI in mice by suppressing SMAD7. The findings suggest that miR-21 is important for cardiac fibroblast activation and fibrosis after MI by acting on TGF-β/Smad7 signaling (Figure 2). The Thum lab established the harmful role of miR-21 in cardiac fibrosis [65]. In cardiac fibroblasts, miR-21 revamps the structure and function of failing mice hearts by modulating the ERK-MAP kinase signaling pathway via inhibition of Spry1 [65]. In vivo blocking of miR-21 by antagomir will lower ERK-MAP kinase activity, repress fibrosis and enhance cardiac function [65]. 

#### 3.2.3. Immune Cells 

MI gives rise to a heightened immune response beginning with an acute pro-inflammatory response, which is taken over by an anti-inflammatory reparative state. The goal of miRNA therapeutics is to weaken the initial inflammatory response and promote the reparative phase. 

One factor that governs the advancement and degree of tissue remodeling is the buildup of pro-inflammatory cytokines. The severe inflammation initiated by damage-associated molecular patterns (DAMP) in macrophages explains the development of cardiac dysfunction and remodeling. Thus, a therapy that successfully blocks this process could decrease MI size and enhance cardiac function. miR-21 mimic given to monocytes macrophages in mice reduced inflammatory cytokine expression by targeting KBTBD7 and inhibiting P38 and NF-kB signaling in myocardium post-MI (Figure 2) [96]. miR-21 fine-tunes the mechanisms involved in inflammation triggered by MI.

While acting on different cells in the heart, both miR-1 and miR-21 have harmful roles in the progression of MI. To date, more studies are needed to support the clinical translation of miRNA therapies to treat post-MI complications. 

### 3.3. Studies Integrating mRNA Expression Data from Single-Cell with miRNAs Evident in IR Injury 

For the past decade, high throughput single-cell RNA sequencing (scRNAseq) technologies can generate meaningful mRNA expression profiles for cells. The purpose of scRNseq is to understand the complex role of cells in disease pathology and propose new therapeutic targets. However, miRNAs cannot be captured by single-cell gene expression assays and thus, studied to the same degree. An effective workaround method, premises of miReact software, infers miRNA activity estimates from scRNAseq data by relying on their predefined binding sequence motifs and downstream genes [97].

The advances to derive cell-specific miRNA activity in single-cell data have unlocked the potential to study rare cell types. Per analysis of mouse and human scRNA data, miR-1 activity was specific to cardiac muscle cells [97] and, this was consistent with findings in bulk RNA sequencing data sets and the literature [98]. miR-1 specificity to cardiac cells has the potential to aid in reducing the disease burden. However, this technique is still in its infancy, and more research needs to be performed. 

## 4. Therapeutic Potential of miRNAs in IR Injury

Over the years, experimental evidence supports miRNAs to have cell-specific regulatory roles in cardiac pathophysiology. However, the clinical translational value of miRNAs as therapeutic targets in cardiovascular disease is yet to be determined. 

### 4.1. Modes of miRNA Therapy Delivery

miRNAs control gene expression in up to 90% of the human genome by directly binding to their target genes, mRNA [99]. The expression of miRNAs not only differs between healthy and IR injured hearts but is also dependent on the cell types involved. miRNA therapy needs to involve a delivery system that is effective and also, specific to cell types in the heart. Successful delivery of miRNAs is dependent on overcoming several challenges: intrinsic instability of miRNAs in circulation, off-target effects, and poor distribution [100,101]. miRNAs are compact, hydrophilic molecules that can be administered intravenously or subcutaneously [102]. Yet, the clinical applicability of miRNAs is limited because these single-stranded, open-ended molecules are susceptible to enzymatic degradation or renal excretion [103]. 

A well-established way to silence miRNAs in disease models is to utilize classical antagomirs [104]. Antagomirs, single-stranded RNA inactivator molecules, work by hybridizing to target miRNA via complementary base pairing. In order to have an effect, antagomirs must have key chemistry properties: cell permeability, slow excretion rate, stability in an animal’s body, and interact with miRNA with great specificity and affinity [105,106,107]. With existing technology, locked nucleic acids (LNA) or 2′-O-methyl group (OME) are popular options that chemically modify miRNAs, increasing their stability [15,104]. Upon cellular uptake, these molecules can cause significant knockdown of miRNAs and effectively resolve experimentally induced cardiac pathology [65].

In contrast to antagomirs, synthetic RNA duplexes called agomirs can be used to mimic the endogenous functions of a particular miRNA. Similar to antagomirs, agomirs need to be chemically modified to enhance stability and cellular uptake. The strand that is identical to miRNA of interest is the “guide,” while the strand that is modified with cholesterol is the “passenger” [104]. These molecular mimics effectively restore low levels of miRNA driven by pathology, but problematic because high levels of that miRNA accumulate in off-target tissues [104]. 

Viral vectors, delivery vehicles for miRNAs, are a good way to increase stability during transport, and viral capsids can be altered to target specific tissues [102]. Adeno-associated viruses (AAV), which continuously express the miRNA of interest, have high specificity towards the heart and meet safety standards in clinical gene therapy trials [104]. However, AAV has potential downsides such as unwanted immune activation and incorporation of the virus into the host genome [102]. Other forms of delivery, such as liposomes, lipid-based vectors, protect the miRNA from enzymatic degradation [103]. An enticing option is exosomes, natural carriers of miRNAs because these can deliver miRNA to specific types of cells by receptor-mediated binding and also quickly taken into cells to minimize off-target effects [108]. While other delivery strategies such as nanoparticles [109], “passive-drug targeting” [103], and mesenchymal stem cell-derived extracellular vesicles (MSC-EV) [110] are showing promising results, more research is needed for clinical use.

### 4.2. Potential Benefits of Delivering miRNA Therapies Post-MI

Increasingly, CVD-related research has focused on discovering various cardioprotective interventions that target IR injury associated with atherosclerosis and MI. The targets used for cardioprotective interventions come from mechanistic studies conducted in mice and humans. miR-1 and miR-21 are potential molecular targets or mediators of cardioprotection against IR injury. This section aims to briefly outline the potential role of miR-1 and miR-21 in therapeutic approaches, including the delivery of miR mimics. 

Yin et al. found that mice subjected to cytoprotective heat shock (HS) can upregulate miR-1 and miR-21 in the heart [62]. miRs isolated from HS mice and injected into non-HS mice significantly reduced the infarct size following IR injury. Similarly, the chemically synthesized exogenous miR-21 was cardioprotective [62]. However, miR-21 induced protection stopped when mice were co-treated with miR-21 inhibitor [62]. The use of endogenous miRs as therapy is favorable over other exogenous agents for several reasons. Firstly, endogenous miRs are natural products, thus non-toxic to cells. Secondly, natural conditions (e.g., hyperthermia) can induce endogenous miRs in vivo. Lastly, miRs can easily move across sub-cellular structures due to their small size. Therefore, the precise role of endogenous miRs in the heart may serve as a cardioprotective agent in patients that develop advanced atherosclerosis and subsequent MI. 

As mentioned previously, miR-based therapy can target different cell types in the heart and potentially protect against MI. Bejerano et al. explored whether high levels of miR-21 transcript in macrophage-enriched regions of the infarcted heart could switch their phenotype from an inflammatory (M1) to a reparative (M2) subsequently resolving inflammation and promoting repair in the heart [111]. The nanoparticle delivery of miR-21 mimic to cardiac macrophages improved myocardial remodeling after MI, shown by high angiogenesis, low hypertrophy, fibrosis, and apoptosis [111]. In this study, the laser capture microdissection (LCM) enabled to research macrophages in their natural microenvironment without the need for in vitro cell culture or processing studies [111]. Thus, the delivery of miR mimic, when used with approaches such as LCM, is crucial for evaluating changes in cell phenotypes following cardiac injury. 

While extensive research has shown associations between miRs and IR injury, their therapeutic potential as targets is inconclusive. The majority of the studies, as highlighted in Table 1, do not have a clear acceptance of the role of miR-1 and miR-21 in IR injured hearts. Possibly, the changes in expression of miRs are also dependent on the type of cells involved in IR injury. Thus, a more informed view of these two miRs would be possible with next-generation therapeutic and predictive approaches. To date, whether miR-1 and mR-21 studies will be translated to clinical application is unresolved but continues to be promising. 

## 5. Concluding Remarks

miRNAs are powerful regulators either beneficial or harmful to acute myocardial IR injury and related cardiac diseases. The complex modulatory roles of miR-1 and miR-21 may be heavily dependent on the types of cells involved in each cardiac disease. In order to effectively translate miRNA-based cardiovascular therapies to the clinic, more concrete knowledge of mechanisms of miRNA effects on each cardiac cell type has to be clearly elucidated. Although limited by technology, the focus to design cell-specific miRNAs is emerging as effective methods to treat cardiovascular disease. However, clinical translation of these therapies will require better administration protocols, cell-specific delivery, and additional prognostic models.

## Data Availability

Not applicable.

## Abbreviations

MI	myocardial infarction
PCI	percutaneous coronary intervention
IR	ischemia-reperfusion
miRNAs	microRNAs
miR-1	miRNA-1
miR-21	miRNA-21
CVD	cardiovascular disease
Cx43	connexin 43
Irx5	iroquois homeobox domain 5
KCND2	potassium voltage-gated channel subfamily D member 2
EPCs	endothelial progenitor cells
ROS	reactive oxygen species
PDCD4	programmed cell death 4
scRNAseq	single-cell RNA sequencing
M1	pro-inflammatory macrophage
M2	anti-inflammatory macrophage
PKCε	protein kinase C epsilon
TGF-β	transforming growth factor-β
SMADs	decapentaplegic homologs
TGFβIII	TGF-β receptor III
DAMP	damage-associated molecular patterns
LNA	locked nucleic acids
OME	2′-O-methyl group
AAV	adeno-associated viruses
MSC-EV	mesenchymal stem cell-derived extracellular vesicles
miR-133	miRNA-133
